# Functional analysis of *CTLA4* promoter variant and its possible implication in colorectal cancer immunotherapy

**DOI:** 10.3389/fmed.2023.1160368

**Published:** 2023-08-03

**Authors:** Mariana Angulo-Aguado, Sarah Orjuela-Amarillo, Julián Francisco Mora-Jácome, Lea Paloma Córdoba, Antonio Gallego-Ortiz, Cristian Camilo Gaviria-Sabogal, Nora Contreras, Carlos Figueroa, Oscar Ortega-Recalde, Adrien Morel, Dora Janeth Fonseca-Mendoza

**Affiliations:** ^1^Universidad Del Rosario, School of Medicine and Health Sciences, Center for Research in Genetics and Genomics (CIGGUR), Institute of Translational Medicine (IMT), Bogotá, Colombia; ^2^Departamento de Coloproctología, Hospital Universitario Mayor-Méderi, Universidad del Rosario, Bogotá, Colombia

**Keywords:** colorectal cancer, *CTLA4*, immune tumoral evasion, immunotherapy, biomarker

## Abstract

**Background:**

Colorectal cancer (CRC) is a prevalent cancer, ranking as the third most common. Recent advances in our understanding of the molecular causes of this disease have highlighted the crucial role of tumor immune evasion in its initiation and progression. *CTLA4*, a receptor that acts as a negative regulator of T cell responses, plays a pivotal role in this process, and genetic variations in *CTLA4* have been linked to CRC susceptibility, prognosis, and response to therapy.

**Methods:**

We conducted a case-control study involving 98 CRC patients and 424 controls. We genotyped the *CTLA4* c.-319C > T variant (rs5742909) and performed an association analysis by comparing allele frequencies between the patients and controls. To assess the potential functional impact of this variant, we first performed an *In Silico* analysis of transcription factor binding sites using Genomatix. Finally, to validate our findings, we conducted a luciferase reporter gene assay using different cell lines and an electrophoretic mobility shift assay (EMSA).

**Results:**

The case-control association analysis revealed a significant association between *CTLA4* c.-319C > T and CRC susceptibility (*p* = 0.023; OR 1.89; 95% CI = 1.11–3.23). Genomatix analysis identified LEF1 and TCF7 transcription factors as specific binders to *CTLA4* c.-319C. The reporter gene assay demonstrated notable differences in luciferase activity between the c.-319 C and T alleles in COS-7, HCT116, and Jurkat cell lines. EMSA analysis showed differences in TCF7 interaction with the *CTLA4* C and T alleles.

**Conclusion:**

*CTLA4* c.-319C > T is associated with CRC susceptibility. Based on our functional validation results, we proposed that *CTLA4* c.-319C > T alters gene expression at the transcriptional level, triggering a stronger negative regulation of T-cells and immune tumoral evasion.

## Introduction

Colorectal cancer (CRC) is the third most incident type of cancer and the second leading cause of death among cancer patients worldwide (*GLOBOCAN source*, 2020–2021). The pathogenesis of this disease is complex, heterogeneous and is influenced by several factors, including lifestyle, environment, and genetics. Genetic susceptibility is driven by germline variants and the accumulation of somatic mutations that disturb key processes involved in cell cycle and promote tumorigenesis, including tumor suppressor genes, protooncogenes and immunogens (1). Notably, genetic factors have been shown to contribute up to 35% of the etiology of this disease (2).

Increasing evidence in cancer biology highlights the significance of the immunological landscape and tumor immune evasion as one of the hallmarks of cancer. Several genes, such as *NKG2D, CD28, TNFRSF4, CTLA4, CD80, CD86*, and *PD-1*, have been associated with this immune response. Currently, conventional treatment approaches for CRC include surgery, chemotherapy and radiotherapy (3). More recently, biological therapies based on monoclonal antibodies have been approved, representing a promising area of biopharmaceutical research (4). Specific antibodies that block or deactivate immunological checkpoints and induce antitumor immune responses have been developed and are employed in cancer treatment (5). Ipilimumab, an anti-CTLA4 monoclonal antibody, has received regulatory approval from agencies such as the Food and Drug Administration (FDA) and the European Medicines Agency (EMA) for the treatment of cancer treatment, including metastatic CRC). It is primarily used in advanced melanoma treatment and has demonstrated a complete response rate of 19% (6). The CHECKMATE-142 trial aimed to assess the effectiveness and safety of ipilimumab in patients with advanced colorectal cancer. The trial demonstrated promising activity of ipilimumab either alone or in combination with nivolumab, in a subset of patients with microsatellite instable colorectal cancer (7).

The cytotoxic T-lymphocyte antigen-4 (*CTLA4*) gene, also known as cluster of differentiation 152 (CD152), encodes a transmembrane type 1 T cell inhibitory receptor and plays a critical role as an immune checkpoint. This gene belongs to the IgG superfamily and is transiently expressed on activated T cells while constitutively expressed on regulatory T cells. CTLA4 has two regulatory pathways: an intrinsic regulatory pathway involving direct interaction with the TCR-CD3 complex, leading to downstream negative regulation after T-cell receptor activation (8) and an extrinsic, pathway where CTLA4 competes with CD28 for interactions with the CD80 and CD86 ligands (9).

Considering its role in maintaining immunological balance, genetic variants in *CTLA4* have the potential to modify the immune response and, susceptibility to cancer development. A comprehensive meta-analysis conducted by Fang et al. (2018), which included a total of 67 case-control studies, reported the involvement of several *CTL4* SNPs and proposed the utility of rs5742909 as predictive genetic biomarker for cancer predisposition (10). However, the association between rs5742909 and CRC susceptibility is conflicting, and some researchers did not find a significant association (11). Functional characterization of this variant in CRC has not yet been reported.

Molecular variants such as *CTLA4* c.-319C > T (rs5742909) may impact gene expression variability through transcriptional regulation, affecting the binding sites for specific transcription factors. TCF7/LEF1, members of the high-mobility group (HMG) transcription factor family, play a key role in regulating T cell development and differentiation, as well as the Wnt signaling pathway involved in various cellular processes underlying colorectal cancer (12–14). The genetic variant rs5742909 is located within the *CTL4* promoter consensus Tcf/Lef motif, suggesting its potential influence on the binding affinity of Tcf/Lef transcription factors and subsequent gene expression regulation impacting CRC T cells immune surveillance (15).

Immunotherapeutic biomarkers play a crucial role in predicting treatment response and guiding the use of immunotherapies in CRC. Current immunotherapeutic biomarkers in CRC include: high microsatellite instability (MSI-H)/mismatch repair deficient (dMMR) status (7); programmed death-Ligand 1 (PD-L1) expression (16); tumor-infiltrating lymphocytes (TILs) (17) and immune gene signatures that provide information about the tumor’s immune environment and potential response to immunotherapy (18). Despite this repertoire of biomarkers, it is of great interest to incorporate new options that can be easily evaluated in patient blood samples. In this context, this study assessed the association between the *CTLA4* c.-319C > T variant and CRC susceptibility, proposing its potential use as a biomarker for therapeutic response to anti-CTLA4 monoclonal antibody immunotherapy.

## Methods

### Sampling and data collection

This study included 100 patients who attended the Hospital Universitario Mayor Méderi, Bogotá, Colombia. Patients whose biopsy confirmed CRC, and accepted and signed the informed consent form were recruited for this study. The patients were enrolled between July 2017 and December 2021. The inclusion criteria included patients diagnosed by pathology with any type of colorectal cancer, including individuals with metastatic CRC tumors. Genomic data from healthy controls were obtained from the gnomAD public database v2.1.1,^1^ filtering Latin-American and non-cancer individuals. A total of 424 participants met these criteria for a case–control ratio of 1:4.

The sample size was considered according to the value obtained using the formula *n* = Nz2*p(1 − *p)/(α2(N* − *1)* + z2*p(1 − *p)*, accessible in the OpenEpi web tool.^2^ Considering that this study was the first to evaluate the allelic frequency of rs5742909 in the Colombian population, a *p* (sample proportion) of 7.4% was considered, this value corresponds to the minimum allele frequency (MAF) of the polymorphism in the Latin-American population reported in the public database gnomAD v2.1.1 (non-cancer). A confidence level of 99% (*α* = 0.001, *z* = 2.576), and a population finite size *N* = 8,000,000 for Bogotá, the city where the study was conducted, were used for the estimation. The obtained value was *n* = 506, which was approximated to 524 individuals (case and controls) considering possible losses of data.

Sociodemographic and clinical variables of the patients were collected through structured interviews and clinical records by trained healthcare professionals. The variables assessed were sex, age, comorbidities (hypertension, diabetes mellitus, chronic obstructive pulmonary disease, cancer, inflammatory bowel disease, and others), family history of cancer, habits, CRC screening tests, height, weight, age at diagnosis, tumor location, histology, lymphovascular infiltration, pTNM classification, and stage. The study protocol and all procedures were approved by the Ethics Committee of the Universidad del Rosario (CEI – DVN021-000285) and the technical committee of the Hospital Universitario Mayor Méderi. This study adhered to the Declaration of Helsinki guidelines.

### DNA extraction and genotyping

Genomic DNA was obtained from blood samples of 100 patients using the Quick-DNA^™^ Miniprep Plus Kit (Zymo Research). Polymerase chain reaction (PCR) was used to amplify the *CTLA4* promoter region from −500, considering the first ATG as +1, to the end of the first exon. The PCR products were purified and directly sequenced by Sanger sequencing. Primers were designed using Primer-BLAST. The reference sequence used was obtained from the Ensembl database (ENST00000648405.2). Primers and PCR conditions are listed in Supplementary Table S1. Genotyping of the rs5742909 was performed in batches of 25 samples by two independent researchers, genotyping was attempted in 100 individuals and was successful in 98% (*n* = 98). Control genotypes were obtained from the genomes of Latin American non-cancer individuals from the gnomAD public database (see footnote 1) v2.1.1 (non-cancer). The genotype quality for controls ranged from 95 to 100 and the read depth was more than 20X for >95% of variant carriers.

### Population genetic statistics and polymorphism association

Genotypic and allele frequencies, and Hardy–Weinberg equilibrium were determined for the case and control groups. Deviation from HWE was estimated using a chi-square (*χ*^2^) goodness-of-fit test with 1 degree of freedom (1df) using the SNP-Stats software.^3^ For the case–control analysis, genotypes were compared under different genetic models including codominant (C/C vs. C/T vs. T/T) dominant (C/C vs. C/T–T/T) and recessive (C/C-C/T vs. T/T). *χ*^2^tests with 2 degrees of freedom (2df) for codominant and 1df for dominant and recessive models were used to identify statistically significant differences. The best model was selected based on the Akaike information criterion (AIC). Finally, odds ratios (OR), 95%, and confidence interval (CI) values were determined using SNP-Stats. This study was conducted following the Extension for Genetic Association Studies (STREGA) guidelines recommendations (19).

### *In silico* transcription factor binding site analysis and luciferase reporter assay

Potential transcription factor binding sites (TFBS) on *CTLA4* c.-319C promoter region were assessed using Genomatix bioinformatics tools MatInspector (v8.4.2) and MatBase (v11.3).^4^ Values of 0.75 core similarity and 0.70 matrix similarity were set as parameter cutoffs.

### Plasmid constructs

Using genomic DNA obtained from a patient heterozygous for the *CTLA4* c.-319C > T variant, we amplified a region that encompassed the *CTLA4* promoter region from −1 to −575 pb. Forward and reverse primers used in PCR contained *Kpn*I and *Xho*I restriction sites located at the 5’and 3′ ends, respectively. Primers were designed using Primer-BLAST and the sequences are listed in Supplementary Table S1. The PCR products were digested and ligated into the pGL4.22luc2CP/Puro plasmid (#E6771/Promega) following the manufacturer’s instructions. The constructs were sequenced to verify the generation of plasmids carrying *CTLA4* c.-319C and c.-319 T alleles.

The pGEM-T plasmid containing the full-length clone DNA of human *LEF1* (Human lymphoid enhancer-binding factor 1) (Sino Biological Cat:HG12090-G)^5^ was used as a template for cloning the *LEF1* open reading frame (ORF) into the pcDNA^™^3.1/V5-His TOPO^™^ TA Expression Kit (ThermoFisher Scientific cat K480001).^6^ The pcDNA3-HA-TCF1 plasmid, containing TCF-1 (T-Cell Factor 1), known also as TCF7 (transcription factor 7), was a kind gift from Dr. Kai Ge (Addgene plasmid # 40620; http://n2t.net/addgene:40620;RRID:Addgene_40620). *LEF1*, *TCF7* full-length cDNA was cloned into the pcDNA^™^3.1/V5-His TOPO^™^ TA Expression Kit, according to the manufacturer’s recommendations. Subcloning was verified using Sanger sequencing.

### Cell culture and luciferase reporter gene assay

Three cell lines were used for the reporter gene assay considering the variability in LEF1 and TCF7 regulation effects depending on the cell type and available co-factors. Cell lines used HCT116, COS-7 and Jurkat are all well-know and have been extensively used in functional validation approaches. We chose these cell lines for their application in studies of colorectal cancer biology such as human colorectal carcinoma cell line, HCT116; Jurkat is a human T-Cell leukemia cell line extensively used in immunology research and COS-7 cells commonly used for protein expression that require high transfection efficiency. HCT116 and COS-7 are adherent while Jurkat are suspension cells.^7^ HCT116 and COS-7 cell lines were grown in Dulbecco’s Modified Eagle’s medium (DMEM) (see footnote 6) supplemented with 10% fetal bovine serum and 100 U/mL penicillin/streptomycin at 37°C in 5% CO_2_. Jurkat cell line were cultured in RPMI 1640 medium (see footnote 6) supplemented with 10% fetal bovine serum, 1 mM sodium pyruvate and 100 U/mL penicillin at 37°C in 5% CO_2_. HCT116 and COS-7 cells were seeded at 60,000 cells per well in 24-well plates, in triplicate or quintuplicate for each condition. Cells were co-transfected for 48 h using 750 ng of *CTLA4* promoter (C-allele or T-allele) expressing luciferase reporter, 10 ng of the pRL renilla luciferase control reporter vector (Promega, Madison, WI, United States), and 250 ng of the LEF1 or TCF7 expression vector, using Lipofectamine^™^ 3000 transfection reagent (Thermo Scientific) according to the manufacturer’s protocol. Jurkat cells were nucleofected (24 h) with 4D nucleofector using Lonza^™^ nucleofector SE cell line kit (PBC1-00675). In both procedures, empty reporter vectors were included as negative controls. After transfection, we assessed promoter activity using the Dual-Luciferase Reporter Assay System (DLR) (Promega, Madison, WI, United States). Luciferase values were presented as relative luciferase units (RLU) and normalized to the wild-type *CTLA4* promoter. RLU are a measurement used to quantify the activity of the luciferase enzyme and the values are obtained by measuring the amount of light emitted by luciferase in cell models. Since the light output is directly proportional to luciferase activity, RLU provides a relative measure of promoter activity.

After testing normality using the Shapiro–Wilk test, Student’s *t*-test (2-tailed, non-paired *t*-test) was used to determine statistical differences; otherwise, the Mann–Whitney test was implemented. Statistical analyses were performed using Jamovi software v1.81 (The jamovi project (2021). jamovi. (Version 1.8) [Computer Software]. Retrieved from https://www.jamovi.org) and *p*-values <0.05 were considered statistically significant.

### Electrophoretic mobility shift assay

Custom double-stranded oligonucleotides 5’end-labeled with biotin and oligonucleotides containing the TCF7 consensus sequence for human *CTLA4* c.-319 C or T promoter were used in the EMSA procedure. The sequences used are listed in Supplementary Table S1. COS-7 cells were transfected with the pcDNA TCF7 vector. After 48 h, nuclear proteins were extracted using the NE-PER Nuclear and Cytoplasmic Extraction Reagents (Catalog number 78833, ThermoScientific). EMSA was assessed using the LightShift Chemiluminescent EMSA Kit following the manufacturer’s instructions. Binding reactions were performed using 10 fmol of double-strand biotin-oligonucleotide, 3 ug of nuclear extract 10 mMol HEPES, 5 mmol DTT, 50 mMol NaCl, 10 mMol KCl, 1.5 mMol EDTA, 15%, 1 mg/mL BSA, 0.5 mMol PMSF, and 2 μg poly (dI:dC). The reactions were incubated for 15 min on ice before adding biotin-labeled DNA and then for 1 h at room temperature. Electrophoresis of the DNA-protein complex was performed on 6% polyacrylamide and 3% glycerol gels at 80 V in TBE 0.5× buffer and transferred to a nylon membrane for biotin-labeled DNA detection using streptavidin-horseradish conjugate and the chemiluminescent reagent contained in the EMSA Kit.

## Results

### Clinical and demographic characteristics

This study analyzed a total of 98 cases (Table 1). This group was similarly distributed between males (51.0%, *n* = 50) and females (48.9%, *n* = 48), with the majority being over 50 years old (89.8%, *n* = 88). Body mass index (BMI) was normal (18.5–24.9 kg/m^2^) in 43.6% of patients (*n* = 38). The most prevalent comorbidities were hypertension (57.1%, *n* = 56), type 2 diabetes mellitus (21.4%, *n* = 21), and chronic obstructive pulmonary disease (7.1%, *n* = 7). 13.2% of patients (*n* = 13) had no comorbidities and 65.3% (*n* = 64) exhibited other types of comorbidities. A family history of cancer was reported in 70% (*n* = 66) of cases of which familial adenomatous polyposis (FAP) syndrome was observed in 3.1% (*n* = 3), CRC history was present in 5.3% (*n* = 5) and 61.7% (*n* = 58) of cases had a family history of other types of cancer.

**Table 1:** Sociodemographic characteristics of CRC patients.

Clinicopathologic feature	CRC patients	CRC patients (%)
Sex (*n* = 98)		
Female	50	51.0
Male	48	49.0
Age (*n* = 98)		
<50 Years	10	10.2
>50 Years	88	89.8
BMI (*n* = 87)		
<18.5 Kg/m^2^	1	1.1
18.5–24.9 Kg/m^2^	38	43.7
25–29.9 Kg/m^2^	35	40.2
30–34.9 Kg/m^2^	8	9.2
35–39.9 Kg/m^2^	5	5.7
>40 Kg/m^2^	0	0.0
Comorbidities (*n* = 98)		
None	13	13.3
Hypertension	56	57.1
Diabetes	21	21.4
COPD	7	7.1
Others	69	70.4
Family history (*n* = 94)		
FAP	3	3.2
CRC	5	5.3
Another type of cancer	58	61.7
None	28	29.8
Red meat consumption (*n* = 85)		
≥3 days	52	61.2
<3 days	33	38.8
Dietary fiber (*n* = 85)		
≥2 times a day	51	60.0
<2 times a day	34	40.0
Tobacco smoking (*n* = 88)		
Active	2	2.3
Non-active	46	52.3
Non-smoker	40	45.5
Alcohol intake (*n* = 87)		
Active	5	5.7
Non-active	30	34.5
Non-consumer	52	59.8
Physical activity (*n* = 72)		
Active	45	62.5
Non-active	27	37.5
Colonoscopy screening (*n* = 85)		
Previous screening	13	15.3
Non-screened	72	84.7
Location (*n* = 98)		
Right colon	47	48.0
Transverse colon	3	3.1
Left colon	2	2.0
Sigmoid colon	35	35.7
Rectum	19	19.4
Whole colon	1	1.0
Histology (*n* = 98)		
Well differentiated adenoma	20	20.4
Moderately differentiated adenoma	68	69.4
Poorly differentiated adenoma	7	7.1
Infiltrating adenocarcinoma	11	11.2
Mucinous adenocarcinoma	50	51.0
Lymphovascular infiltration (*n* = 97)		
Present	56	57.7
Absent	41	42.3
Metastasis (*n* = 97)		
Yes	22	22.7
No	75	77.3
Stage (*n* = 98)		
0	2	2.0
I	13	13.3
II	34	34.7
III	32	32.7
IV	17	17.3
Surgery performed (*n* = 98)		
Right hemicolectomy	46	46.9
Left hemicolectomy	6	6.1
Sigmoidectomy	25	25.5
Proctosigmoidectomy	3	3.1
Colectomy	4	4.1
Anterior resection	11	11.2
Other	4	4.1
Relapse (*n* = 92)		
Yes	12	13.0
No	80	87.0

BMI, body mass index; COPD, chronic obstructive pulmonary disease; CRC, colorectal cancer; FAP, familial adenomatous polyposis.

Regarding diagnosis, 17.3% of patients (*n* = 17) had stage IV cancer, 32.6% (*n* = 32) stage III, 34.6% (*n* = 34) stage II, 13.2% (*n* = 13) stage I and 2% (*n* = 2) stage 0. Of these, 22.6% had metastasis (*n* = 22) and 57.7% (*n* = 56) presented lymphovascular infiltration. 12 patients (13.0%) had a relapse defined as local, regional, and distant metastatic recurrence after a disease-free period (20). The values obtained were calculated based on the sample size of each variable.

### Genetic statistics and association analysis

The *CTLA4* c.-319C genotype and allele frequencies were determined for 98 cases and 424 controls. The global genotype frequencies were 83.5% (436/522) for CC, 16.3% (85/522) for CT and 0.2% (1/522) for TT, with allelic frequencies of 91.75 and 8.25% for C and T alleles, respectively. This variant was found to be in HWE (*p* = 0.24). Genotypic and allelic frequencies according to case-control status are presented in Table 2. According to the minimum AIC value, the best genetic model was dominant (AIC = 503; *p* = 0.023). Association analysis for this model identified a statistically significant association between *CTLA4* c.-319C > T and CRC development (*p* = 0.023; OR 1.89; 95% CI = 1.11–3.23). The results of the codominant, dominant, and recessive association analyses are presented in Supplementary Table S2.

**Table 2:** Allelic and genotypic frequencies for case and controls.

Gen	SNP	Allele frequency controls		Allele frequency cases		Genotype controls			Genotype cases		
*CTL4*	rs5742909	WT	Alt	WT	Alt	WT/WT	WT/Alt	Alt/Alt	WT/WT	WT/Alt	Alt/Alt
		0.93	0.07	0.88	0.12	0.85	0.14	0.00	0.76	0.24	0

Alt, alternative allele; *CTL4*, cytotoxic T-lymphocyte antigen 4; SNP, single nucleotide polymorphism; WT, wild type.

### *In silico* binding site prediction and promoter activity

**Figure 1:**
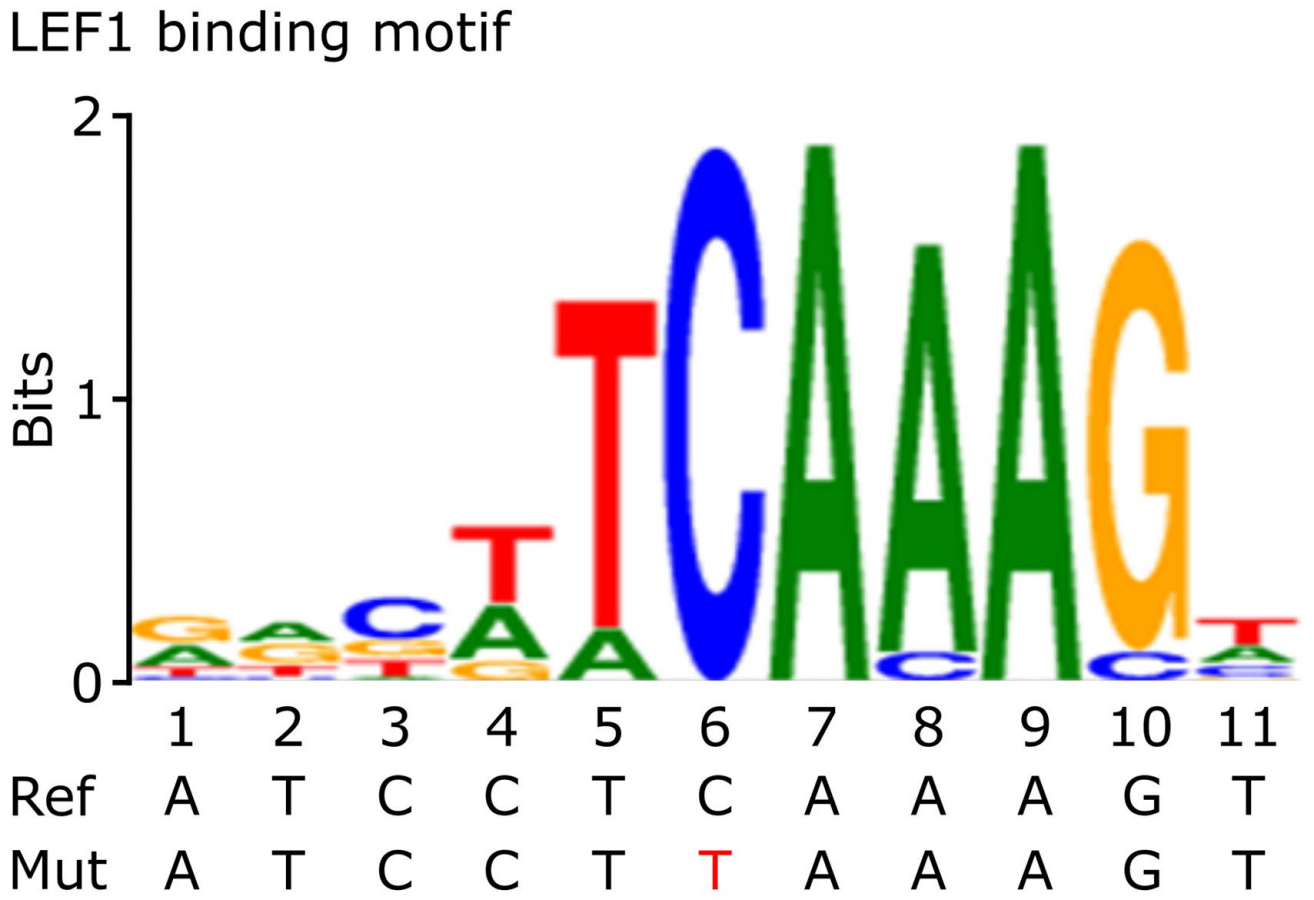
Consensus sequence of LEF1. This figure shows the Consensus sequence of LEF1 and the localization of the *CTLA4* promoter variant (in red). This figure was adapted from Genomatix bioinformatics tools MatInspector (v8.4.2) and MatBase (v11.3). Precigen Bioinformatics Germany GmbH, license GEMV-0112 (http://mygga.genomatix.de/).

To investigate the functional and regulatory role of the rs5742909 promoter variant, we first searched for potential TFBS using the Genomatix software. This *in silico* approach predicted that the LEF1 and TCF7 transcription factors bind to the *CTLA4* c.-319 promoter region. The bioinformatics platform determined that LEF1 and TCF7 bind to the sequence 5´-agatccTCAAAGtgaac-3′ and that the variant *CTLA4* c.-319C > T is located in the core consensus motif (Figure 1). These results suggest that the rs5742909 variant could disrupt the *CTLA4* promoter binding site for LEF1/TCF7.

**Figure 2:**
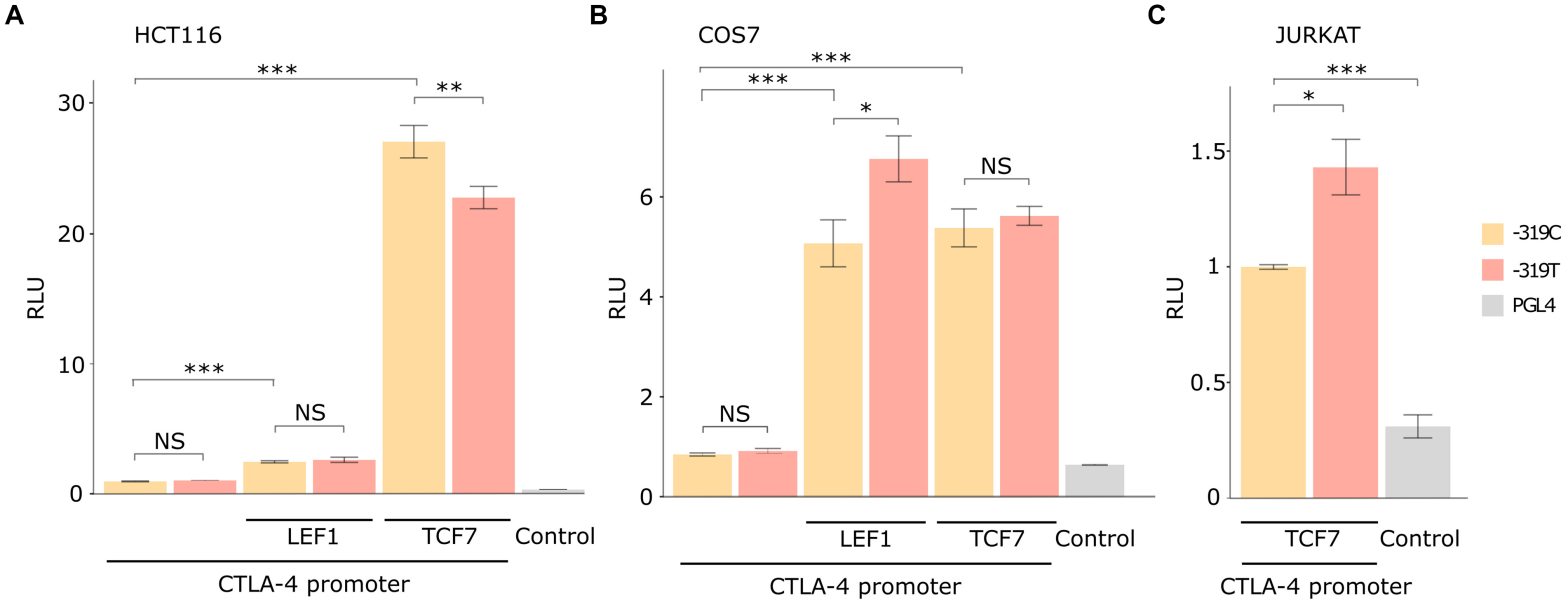
Luciferase reporter assay results in different cellular models. The data are presented after normalizing the transfection efficiency using the Renilla luciferase reporter gene, and RLU values were normalized to each WT control. Total replicate numbers were **(A)** HCT116 cell line (*n* = 9), **(B)** Jurkat cell line (*n* = 6), and **(C)** COS-7 cell line (*n* = 15). Error bars indicate standard deviation. *t*-test or Mann–Whitney U-test results considered statistically significant ***p < 0.001, **p < 0.01, and *p < 0.05, NS (Non statistical significance).

To test this hypothesis, we conducted two *in vitro* assays: a luciferase reporter assay and EMSA. Luciferase reporter gene assay indicated that the *CTLA4* promoter is transactivated by LEF1 and TCF7 transcription factors in COS-7, HCT116, and Jurkat cell lines (*p* < 0.001). Significant differences in luciferase activity among the c.-319C or T alleles were observed for the three cell lines (Figure 2). Notably, these effects on transactivation varied according to the cell line and the transcription factor (Figure 2). For example, the T allele decreased RLU by 20% for TCF7 transactivation in HCT116 cells (22.8 ± 0.8 vs. 27.1 ± 1.2) when compared with wild-type (*p* = 0.008). No significant differences were observed for LEF1 in this cell line (Figure 2A). In contrast, the alternative allele (T) increased RLU by 30% for LEF1 in COS-7 cells (6.76 ± 0.4 vs. 5.07 ± 0.4) when compared with the C allele (*p* = 0.015). Similarly, a significant promoter activity enhancement was found for TCF7 in Jurkat cells (*p* = 0.01; Figure 2B). The luciferase *CTLA4* promoter activity was significantly higher when compared to the pGL4 empty vector (*p* < 0.001) for all cell lines (Figure 2C; Supplementary Table S3).

### DNA-specific binding analyses

**Figure 3:**
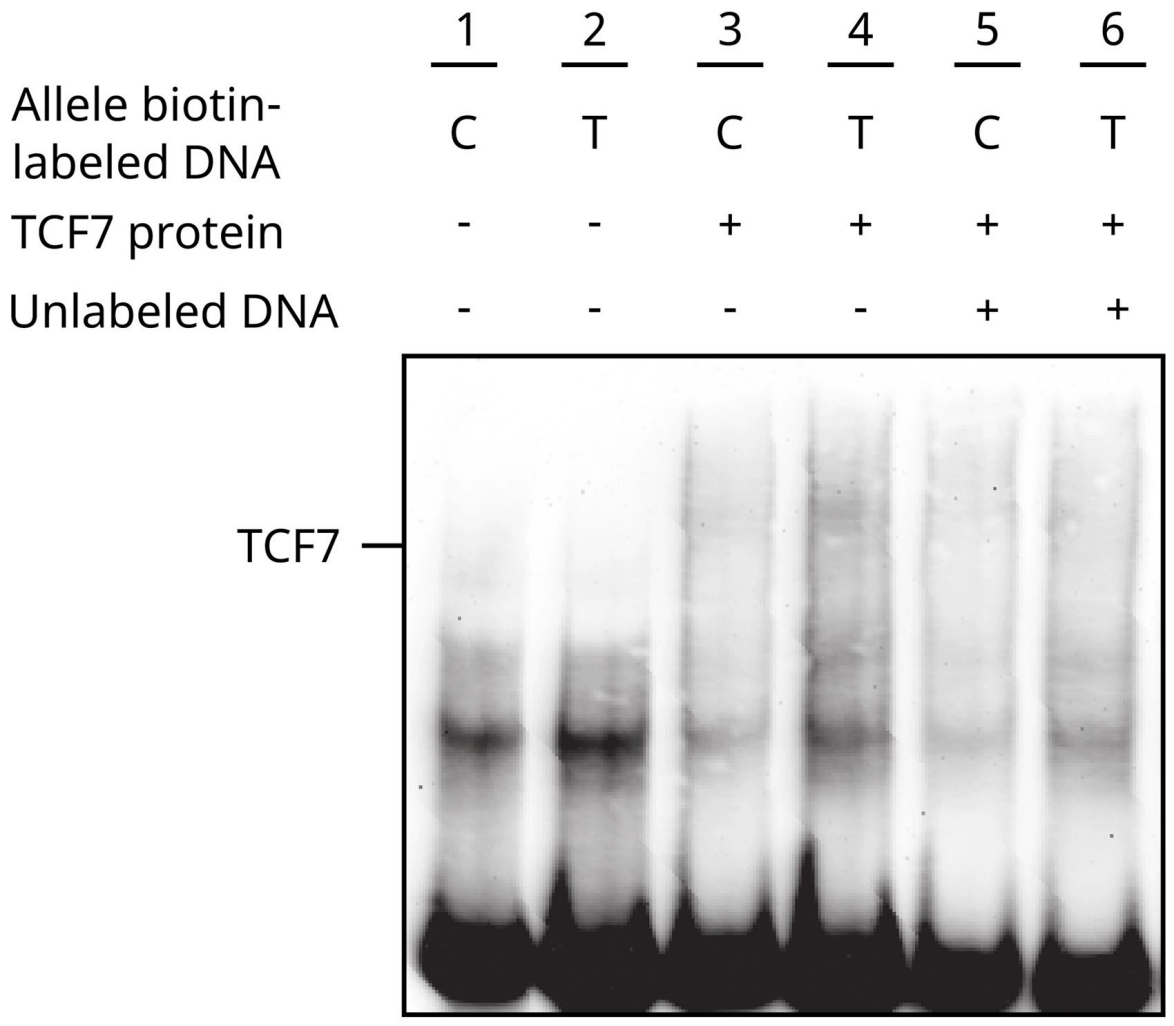
*CTLA4*-TCF7 interaction assessment through EMSA. Line #1–2 Control -319 C/T allele biotin-labeled DNA, #3–4 -319 C/T allele biotin-labeled DNA + transfected TCF7 cell nuclear extracts, #5–6 binding competition analysis for -319 C/T allele biotin-labeled DNA with unlabeled C allele oligonucleotides.

The EMSA assay demonstrated the interaction of the TCF7 transcription factor with biotin-labeled oligonucleotides of the *CTLA4* promoter. The affinity and strength of binding revealed an increased band intensity for the T allele in the nuclear extracts of transfected TCF7 cells. A similar pattern of band enrichment was observed in the nuclear extracts of non-transfected cells (Figure 3). These results suggested that the binding was specific, considering that there was competition by unlabeled DNA, where band intensity was meaningfully reduced.

## Discussion

CRC is a multifactorial disease with both environmental and genetic factors contributing to its pathogenesis. As illustrated in Table 1, the majority of CRC patients (89.8%) were over 50 years old, which is consistent with the age cut-off defining late-onset colorectal cancer (21). Most cases of the disease are sporadic and approximately 25% of CRC cases have a positive family history (22). However, only 5,32% of our patients met these criteria while 61,7% of them had family history of other types of cancer. The World Cancer Research Fund and American Institute of Cancer Research have established obesity, low physical activity, diets rich in high red and processed meat and low in fiber and alcohol intake as main risk factors for CRC development (22). Importantly, the sociodemographic variables taken in CRC cases allow us to identify that 56,4% of patients are overweight or obese, 61,18% consume red meat three or more days per week. Additionally, 86,73% of them have comorbidities with special consideration given to type 2 diabetes and hypertension as well studied risk factors (23, 24). Considering that we used a population database (gnomAD) to obtain controls genomic data to perform the case–control analysis, it was not possible to compare all of these clinical variables among groups.

Among the key features of CRC oncogenesis and progression, immune surveillance escape has gained increasing importance, particularly as a targeted therapy (25). At the cellular level, the immune system initially attempts to eliminate malignant cells via cytotoxic or natural killer (NK) lymphocytes. However, over time, tumors enter an equilibrium phase and display resistance. Finally, the tumor reaches an escape phase, where neoplastic growth, proliferation, and dissemination of cancer cells saturate the immune system (26).

CTLA4 is a negative regulator of T-cell function and modulates the duration and strength of T cell mediated immune responses through several intrinsic and extrinsic mechanisms, triggering anergy and immune tolerance (27, 28). Several studies have reported significant associations between frequent *CTL4* polymorphisms and cancer susceptibility, including CRC (11, 29–35). We identified a positive association between *CTL4* c.-319C > T (rs5742909) and CRC susceptibility (dominant genetic model, *p* = 0.023). This positive association could be related to impairment of the T cell antitumor immune response.

The rs5742909 variant is located in the *CTLA4* promoter region and may alter the DNA binding of transcription factors and impact gene expression via transcriptional regulation. Consistent with our findings, Gibson et al., 2007, used serial *CTLA4* promoter deletions and luciferase reporter assays to identify an essential regulatory region located between −200 and −330 bp, which overlaps with the studied variant. A few specific T-cell transcriptional factors have been identified in the *CTLA4* promoter (36). The interaction of these factors with *CTL4* promoter influences the expression of CTLA-4, which is a critical checkpoint molecule in immune regulation. NFAT (Nuclear Factor of Activated T cells) binding sites in the *CTL4* promoter have been implicated in the induction of CTLA-4 expression upon T-cell activation. Recent studies have demonstrated that NFATC2 promotes the stemness of colorectal cancer stem cells via AJUBA-mediated YAP activation and constitutes a novel therapeutic target (37).

According to our Genomatix *in silico* analysis (Figure 1), the *CTLA4* c.-319C > T variant is located in a core consensus motif (TCAAAG) for LEF1 and TCF7 transcription factors (15, 38). *CTLA4* transcriptional regulation mediated by LEF1/TCF7 is a molecular pathway conserved in multiple T-lineage cells (39). In agreement with these observations, our luciferase reporter assay results demonstrated that LEF1 and TCF7 transcription factors positively activated the *CTLA4* c.-319C promoter in COS-7 and HCT116 cell lines (Figure 2).

It has been reported that LEF1/TCF7 has variable activity and regulation depending on the cell type and available co-factors. Therefore, we used three cell lines to confirm the differences between the two alleles. *CTLA4* T allele has 30% higher promoter activity for LEF1 transactivation on COS-7 cells (Figure 2), in agreement with previous studies in Jurkat T cells models (40, 41). Transactivation regulation by TCF7 was significant in HCT116 and Jurkat cell lines with statistical differences for the c.-319C and T alleles. In the HCT116 cell line, *CTLA4* c.-319 T allele exhibited a diminished promoter activity, whereas for Jurkat cell line, we found the opposite effect (Figure 2). The EMSA assay revealed an increased band intensity for *CTL4* T allele, suggesting an increase in the binding affinity. Furthermore, we confirmed TCF7’s capability to activate or suppress CTLA4 transcriptional activation depending on the C/T alleles and different cellular contexts. In HCT116 cells, the *CTLA4* c.-319 T allele exhibited diminished promoter activity, whereas, in Jurkat cells, we found the opposite effect. We demonstrated TCF7’s heterogeneous role and its context dependent CTLA4 transcriptional regulation. This modulation can be influenced by its interaction with other signaling pathways and transcription factors. TCF7 has been found to interact with the Wnt signaling pathway, β-catenin, and other factors involved in T cell development and differentiation (42). Conversely, TCF7 can interact with other transcriptional regulators, such as Foxp3, to repress *CTL4* gene expression (43). This negative regulation may be relevant in the context of regulatory T cells (Tregs), where CTLA4 expression is crucial for their suppressive function. These interactions with other transcription factors, co-factors, and our identified promoter variant c.-319C > T can modulate the overall impact of TCF7 on *CTL4* gene expression. The exact mechanisms and conditions governing TCF7’s role in CTLA4 transcriptional regulation are still an active area of research, and further studies are needed to fully understand the complexity and context-dependent nature of this regulation. Nevertheless, these findings highlight the importance of considering *CTL4* promoter molecular variants as contributing factors to TCF7’s effect on CTLA4 expression.

Our study identified TCF7 as an important transcription factor involved in abnormal transactivation of the *CTLA4* T allele as evidenced in the non-activated Jurkat cells luciferase assay (Figure 2). The relevance of TCF7 binding consensus region has been demonstrated in EMSA assays using specific inhibitors, such as RNA aptamers (44–46). Our results support these observations, showing a decreased binding between TCF7 and *CTL4* T allele (Figure 3).

*CTLA4* c.-319 CT/TT genotypes have been associated with decreased IL-2 levels, which, in turn, stimulate TCF7 expression, suggesting a potential positive feedback mechanism (47–49). CTLA4 abnormal expression could lead to inappropriate TCF7 regulation affecting molecular pathwyas related to CRC, such as Wnt/β-catenin (47, 50). On the other hand, the enhanced promoter activity of the *CTLA4* T allele could increase CTLA4 mRNA levels and the expression of CTLA4 protein in T-cells affecting proliferation and activation, mitigating the anti-tumoral immune response, and promoting tumoral immune surveillance escape, thus conferring an increased risk of CRC. Some studies have evaluated the relation between rs5742909 and CTLA4 mRNA expression levels, reporting statistically significant higher expression for the T allele (51, 52).

*CTLA4* gene expression has a significant impact in the clinical setting and the c.-319C > T promoter variant might be useful as a potential prognosis or therapeutic biomarker. Overexpression of this gene has been associated with poor prognosis in several tumor types (53). Currently, it is considered a key therapeutic target for melanoma, non-small cell lung cancer and metastatic CRC. Omura et al., 2020 showed that the CTLA4 overexpression in CRC tissue was associated with worse overall survival (HR = 3.86, value of *p* = 0.001) (54). Consistently, Kamal et al., 2021 found significant CTLA4 upregulation in CRC patients compared to healthy volunteers and suggested that it may be used as an independent prognostic biomarker for survival (55). Our functional validation assays suggest that the *CTLA4* c.-319C > T variant modifies the transcriptional regulation of this gene.

In a therapeutic context, CTLA4 protein has been recognized as the target for immunotherapy drugs such as ipilimumab, a monoclonal antibody approved for advanced CRC treatment (34). To date, only a few biomarkers have been applied in the clinical practice to guide therapeutic decisions. Tumor mutational burden (TMB), microsatellite instability (MSI), T cell-inflamed microenvironment, and TGFβ expression profile are candidate biomarkers for CRC, but their analyses are expensive, delayed, and not easily available (56, 57). Recent evidence has indicated that methylation levels of *CTLA4* promoter predict therapeutic response in patients affected by melanoma and clear renal cell carcinoma (58, 59). Collectively, our findings suggest a potential use of this molecular variant as a potential novel biomarker for prognosis and therapeutic response.

### Study limitations

The controls used for the association analysis were obtained from a public genomic database, and therefore, we did not have access to their clinical data. It was not possible to blind the researchers in the genotyping process because the case-control status was known from the beginning. We did not have access to clinical data of patients who declined to participate in our study and it is possibly related to selection bias. Additionally, our findings were not replicated in an independent sample which could have been helpful for reducing possible false positive associations. The potential impact of co-factors such as β-catenin or Groucho were controlled only using three different cell lines, but not directly assessed.

## Conclusion

To our knowledge, this is the first report describing the functional impact of the *CTLA4* c.-319 T allele on TCF7 promoter transactivation in the context of CRC. The fact that predictors based on genotyping could be a promising field in personalized medicine is supported by our findings. However, despite the evidence, more experimental and clinical studies would be necessary to validate its performance, including in CRC patients treated with anti-CTLA4 immunotherapy.

## Data availability statement

The original contributions presented in the study are included in the article/Supplementary material, further inquiries can be directed to the corresponding authors.

## Ethics statement

The study was reviewed and approved by the Ethics Committee of the Universidad del Rosario (CEI – DVN021-000285). The patients/participants provided their written informed consent to participate in this study.

## Author contributions

MA-A, DF-M, OO-R, CF, and AM contributed to conception, project administration, supervision, validation, and design of the study. MA-A and SO-A wrote the first draft of the manuscript and performed the statistical analysis. MA-A, SO-A, and AM conducted the experiments. JM-J, LC, AG-O, CG-S, and NC organized the database and wrote sections of the manuscript. All authors contributed to the article and approved the submitted version.

## Funding

This work was support by Universidad del Rosario (Grant ABN062) and Hospital Universitario Mayor-Mederi (Grant QAN-BG272).

## Conflict of interest

The authors declare that the research was conducted in the absence of any commercial or financial relationships that could be constructed as a potential conflict of interest.

## Publisher’s note

All claims expressed in this article are solely those of the authors and do not necessarily represent those of their affiliated organizations, or those of the publisher, the editors and the reviewers. Any product that may be evaluated in this article, or claim that may be made by its manufacturer, is not guaranteed or endorsed by the publisher.

## Footnotes

^1^ https://gnomad.broadinstitute.org/

^2^ http://www.OpenEpi.com

^3^ https://www.snpstats.net/start.htm

^4^ www.genomatix.de

^5^ www.sinobiological.com

^6^ www.thermofisher.com

^7^https://www.atcc.org/cell-products, https://depmap.org/portal/

## References

[1] Markowitz SD Bertagnolli MM. Molecular origins of cancer: molecular basis of colorectal cancer. N Engl J Med. (2009) 361:2449–60. doi: 10.1056/NEJMra0804588, PMID: 20018966 PMC2843693

[2] Haggar FA Boushey RP. Colorectal cancer epidemiology: incidence, mortality, survival, and risk factors. Clin Colon Rectal Surg. (2009) 22:191–7. doi: 10.1055/s-0029-1242458, PMID: 21037809 PMC2796096

[3] Wolpin BM Mayer RJ. Systemic treatment of colorectal cancer. Gastroenterology. (2008) 134:1296–1310.e1. doi: 10.1053/j.gastro.2008.02.098, PMID: 18471507 PMC2528832

[4] Zahavi D Weiner L. Monoclonal antibodies in cancer therapy. Antibodies. (2020) 9:34. doi: 10.3390/antib9030034, PMID: 32698317 PMC7551545

[5] Aris M Barrio MM. Combining immunotherapy with oncogene-targeted therapy: a new road for melanoma treatment. Front Immunol. (2015) 6:46. doi: 10.3389/fimmu.2015.00046, PMID: 25709607 PMC4321613

[6] Larkin J Chiarion-Sileni V Gonzalez R Grob J-J Rutkowski P Lao CD. Five-year survival with combined nivolumab and ipilimumab in advanced melanoma. N Engl J Med. (2019) 381:1535–46. doi: 10.1056/NEJMoa1910836, PMID: 31562797

[7] André T Lonardi S Wong KYM Lenz H-J Gelsomino F Aglietta M. Nivolumab plus low-dose ipilimumab in previously treated patients with microsatellite instability-high/mismatch repair-deficient metastatic colorectal cancer: 4-year follow-up from CheckMate 142. Ann Oncol. (2022) 33:1052–60. doi: 10.1016/j.annonc.2022.06.008, PMID: 35764271

[8] Hwang K Yoon JH Lee JH Lee S. Recent advances in monoclonal antibody therapy for colorectal cancers. Biomedicine. (2021) 9:39. doi: 10.3390/biomedicines9010039, PMID: 33466394 PMC7824816

[9] Verma N Burns SO Walker LSK Sansom DM. Immune deficiency and autoimmunity in patients with CTLA-4 (CD152) mutations. Clin Exp Immunol. (2017) 190:1–7. doi: 10.1111/cei.12997, PMID: 28600865 PMC5588810

[10] Fang M Huang W Mo D Zhao W Huang R. Association of Five Snps in cytotoxic T-lymphocyte antigen 4 and cancer susceptibility: evidence from 67 studies. Cell Physiol Biochem. (2018) 47:414–27. doi: 10.1159/000489953, PMID: 29794444

[11] Dilmec F Ozgonul A Uzunkoy A Akkafa F. Investigation of CTLA-4 and CD28 gene polymorphisms in a group of Turkish patients with colorectal cancer. Int J Immunogenet. (2008) 35:317–21. doi: 10.1111/j.1744-313X.2008.00782.x, PMID: 18680513

[12] Shan Q Li X Chen X Zeng Z Zhu S Gai K. Tcf1 and Lef1 provide constant supervision to mature CD8+ T cell identity and function by organizing genomic architecture. Nat Commun. (2021) 12:5863. doi: 10.1038/s41467-021-26159-1, PMID: 34615872 PMC8494933

[13] Xing S Gai K Li X Shao P Zeng Z Zhao X. Tcf1 and Lef1 are required for the immunosuppressive function of regulatory T cells. J Exp Med. (2019) 216:847–66. doi: 10.1084/jem.20182010, PMID: 30837262 PMC6446865

[14] Mayer C-D DE L GSM Alsehly F Hoppler S. Diverse LEF/TCF expression in human colorectal cancer correlates with altered Wnt-regulated transcriptome in a meta-analysis of patient biopsies. Genes. (2020) 11:538. doi: 10.3390/genes11050538, PMID: 32403323 PMC7288467

[15] Giese K Amsterdam A Grosschedl R. DNA-binding properties of the HMG domain of the lymphoid-specific transcriptional regulator LEF-1. Genes Dev. (1991) 5:2567–78. doi: 10.1101/gad.5.12b.2567, PMID: 1752444

[16] Payandeh Z Khalili S Somi MH Mard-Soltani M Baghbanzadeh A Hajiasgharzadeh K. PD-1/PD-L1-dependent immune response in colorectal cancer. J Cell Physiol. (2020) 235:5461–75. doi: 10.1002/jcp.29494, PMID: 31960962

[17] Bai Z Zhou Y Ye Z Xiong J Lan H Wang F. Tumor-infiltrating lymphocytes in colorectal cancer: the fundamental indication and application on immunotherapy. Front Immunol. (2022) 12:808964. doi: 10.3389/fimmu.2021.808964, PMID: 35095898 PMC8795622

[18] Soldevilla B Carretero-Puche C Gomez-Lopez G Al-Shahrour F Riesco MC Gil-Calderon B. The correlation between immune subtypes and consensus molecular subtypes in colorectal cancer identifies novel tumour microenvironment profiles, with prognostic and therapeutic implications. Eur J Cancer. (2019) 123:118–29. doi: 10.1016/j.ejca.2019.09.008, PMID: 31678770

[19] Little J Higgins JPT Ioannidis JPA Moher D Gagnon F von Elm E. STrengthening the REporting of genetic association studies (STREGA)—an extension of the STROBE statement. PLoS Med. (2009) 6:e1000022. doi: 10.1371/journal.pmed.1000022, PMID: 19192942 PMC2634792

[20] Xu W He Y Wang Y Li X Young J Ioannidis JPA. Risk factors and risk prediction models for colorectal cancer metastasis and recurrence: an umbrella review of systematic reviews and meta-analyses of observational studies. BMC Med. (2020) 18:172. doi: 10.1186/s12916-020-01618-6, PMID: 32586325 PMC7318747

[21] Patel SG Karlitz JJ Yen T Lieu CH Boland CR. The rising tide of early-onset colorectal cancer: a comprehensive review of epidemiology, clinical features, biology, risk factors, prevention, and early detection. Lancet Gastroenterol Hepatol. (2022) 7:262–74. doi: 10.1016/S2468-1253(21)00426-X, PMID: 35090605

[22] Keum N Giovannucci E. Global burden of colorectal cancer: emerging trends, risk factors and prevention strategies. Nat Rev Gastroenterol Hepatol. (2019) 16:713–32. doi: 10.1038/s41575-019-0189-8, PMID: 31455888

[23] Xuan K Zhao T Sun C Patel AS Liu H Chen X. The association between hypertension and colorectal cancer: a meta-analysis of observational studies. Eur J Cancer Prev. (2021) 30:84–96. doi: 10.1097/CEJ.0000000000000578, PMID: 32039929

[24] Guraya SY. Association of type 2 diabetes mellitus and the risk of colorectal cancer: a meta-analysis and systematic review. World J Gastroenterol. (2015) 21:6026–31. doi: 10.3748/wjg.v21.i19.6026, PMID: 26019469 PMC4438039

[25] Shaukat A Kahi CJ Burke CA Rabeneck L Sauer BG Rex DK. ACG clinical guidelines: colorectal cancer screening 2021. Am J Gastroenterol. (2021) 116:458–79. doi: 10.14309/ajg.0000000000001122, PMID: 33657038

[26] Dunn GP Bruce AT Ikeda H Old LJ Schreiber RD. Cancer immunoediting: from immunosurveillance to tumor escape. Nat Immunol. (2002) 3:991–8. doi: 10.1038/ni1102-991 12407406

[27] Chikuma S. CTLA-4, an essential immune-checkpoint for T-cell activation. Yoshimura A, (Ed.) Emerging concepts targeting immune checkpoints in cancer and autoimmunity. Cham: Springer International Publishing (2017). 99–126 10.1007/82_2017_61 28900679

[28] Egen JG Kuhns MS Allison JP. CTLA-4: new insights into its biological function and use in tumor immunotherapy. Nat Immunol. (2002) 3:611–8. doi: 10.1038/ni0702-611 12087419

[29] Lang C Chen L Li S. Cytotoxic T-lymphocyte Antigen-4 +49G/a polymorphism and susceptibility to pancreatic cancer. DNA Cell Biol. (2012) 31:683–7. doi: 10.1089/dna.2011.1417, PMID: 22011251

[30] Li J Wang W Sun Y Zhu Y. CTLA-4 polymorphisms and predisposition to digestive system malignancies: a meta-analysis of 31 published studies. World J Surg Onc. (2020) 18:55. doi: 10.1186/s12957-020-1806-2, PMID: 32178688 PMC7077159

[31] Zheng J Yu X Jiang L Xiao M Bai B Lu J. Association between the cytotoxic T-lymphocyte antigen 4 +49G> a polymorphism and cancer risk: a meta-analysis. BMC Cancer. (2010) 10:522. doi: 10.1186/1471-2407-10-522, PMID: 20920330 PMC2958938

[32] Abtahi S Izadi Jahromi F Dabbaghmanesh MH Malekzadeh M Ghaderi A. Association between CTLA-4+ 49A > G and –318C > T single-nucleotide polymorphisms and susceptibility to thyroid neoplasm. Endocrine. (2018) 62:159–65. doi: 10.1007/s12020-018-1663-8, PMID: 30078171

[33] Arikan S Gümüş A Küçükhüseyin Ö Coşkun C Turan S Cacina C. The effect of CTLA-4 and CD28 gene variants and circulating protein levels in patients with gastric cancer. Turkish J Biochem. (2017) 42:551–8. doi: 10.1515/tjb-2017-0024

[34] Liu J-N Kong X-S Huang T Wang R Li W Chen Q-F. Clinical implications of aberrant PD-1 and CTLA4 expression for cancer immunity and prognosis: a pan-cancer study. Front Immunol. (2020) 11:2048. doi: 10.3389/fimmu.2020.02048, PMID: 33072070 PMC7539667

[35] Paulsen E-E Kilvaer TK Rakaee M Richardsen E Hald SM Andersen S. CTLA-4 expression in the non-small cell lung cancer patient tumor microenvironment: diverging prognostic impact in primary tumors and lymph node metastases. Cancer Immunol Immunother. (2017) 66:1449–61. doi: 10.1007/s00262-017-2039-2, PMID: 28707078 PMC5645427

[36] Gibson HM Hedgcock CJ Aufiero BM Wilson AJ Hafner MS Tsokos GC. Induction of the CTLA-4 gene in human lymphocytes is dependent on NFAT binding the proximal promoter. J Immunol. (2007) 179:3831–40. doi: 10.4049/jimmunol.179.6.3831, PMID: 17785820 PMC4290020

[37] Lang T Ding X Kong L Zhou X Zhang Z Ju H. NFATC2 is a novel therapeutic target for colorectal cancer stem cells. Onco Targets Ther. (2018) 11:6911–24. doi: 10.2147/OTT.S169129, PMID: 30410349 PMC6199214

[38] Grosschedl R Giese K Pagel J. HMG domain proteins: architectural elements in the assembly of nucleoprotein structures. Trends Genet. (1994) 10:94–100. doi: 10.1016/0168-9525(94)90232-1, PMID: 8178371

[39] Li F Zhao X Zhang Y Shao P Ma X Paradee WJ. TFH cells depend on Tcf1-intrinsic HDAC activity to suppress CTLA4 and guard B-cell help function. Proc Natl Acad Sci. (2021) 118:e2014562118. doi: 10.1073/pnas.2014562118, PMID: 33372138 PMC7812797

[40] Wang XB Zhao X Giscombe R Lefvert AK. A CTLA-4 gene polymorphism at position −318 in the promoter region affects the expression of protein. Genes Immun. (2002) 3:233–4. doi: 10.1038/sj.gene.6363869 12058260

[41] Chistiakov DA Savostanov KV Turakulov RI Efremov IA Demurov LM. Genetic analysis and functional evaluation of the C/T(−318) and a/G(−1661) polymorphisms of the CTLA-4 gene in patients affected with graves’ disease. Clin Immunol. (2006) 118:233–42. doi: 10.1016/j.clim.2005.09.017, PMID: 16297665

[42] Cadigan KM Waterman ML. TCF/LEFs and Wnt signaling in the nucleus. Cold Spring Harb Perspect Biol. (2012) 4:a007906. doi: 10.1101/cshperspect.a007906, PMID: 23024173 PMC3536346

[43] van der Veeken J Glasner A Zhong Y Hu W Wang Z-M Bou-Puerto R. The transcription factor Foxp3 shapes regulatory T cell identity by tuning the activity of trans-acting intermediaries. Immunity. (2020) 53:971–984.e5. doi: 10.1016/j.immuni.2020.10.010 33176163 PMC8363055

[44] Park J Schlederer M Schreiber M Ice R Merkel O Bilban M. AF1q is a novel TCF7 co-factor which activates CD44 and promotes breast cancer metastasis. Oncotarget. (2015) 6:20697–710. doi: 10.18632/oncotarget.4136, PMID: 26079538 PMC4653036

[45] Beisner J Teltschik Z Ostaff MJ Tiemessen MM Staal FJT Wang G. TCF-1-mediated Wnt signaling regulates Paneth cell innate immune defense effectors HD-5 and -6: implications for Crohn’s disease. Am J Physiol Gastrointest Liver Physiol. (2014) 307:G487–98. doi: 10.1152/ajpgi.00347.2013 24994854

[46] Park MW Choi KH Jeong S. Inhibition of the DNA binding by the TCF-1 binding RNA aptamer. Biochem Biophys Res Commun. (2005) 330:11–7. doi: 10.1016/j.bbrc.2005.02.119, PMID: 15781225

[47] Willinger T Freeman T Herbert M Hasegawa H McMichael AJ Callan MFC. Human naive CD8 T cells Down-regulate expression of the WNT pathway transcription factors lymphoid enhancer binding factor 1 and transcription factor 7 (T cell Factor-1) following antigen encounter in vitro and in vivo. J Immunol. (2006) 176:1439–46. doi: 10.4049/jimmunol.176.3.1439, PMID: 16424171

[48] Liu K Catalfamo M Li Y Henkart PA Weng N. IL-15 mimics T cell receptor crosslinking in the induction of cellular proliferation, gene expression, and cytotoxicity in CD8^+^ memory T cells. Proc Natl Acad Sci U S A. (2002) 99:6192–7. doi: 10.1073/pnas.092675799, PMID: 11972069 PMC122925

[49] Wang J Wang Z Tan T. Association of CTLA-4, TNF alpha and IL 10 polymorphisms with susceptibility to hepatocellular carcinoma. Scand J Immunol. (2019) 90:e12819. doi: 10.1111/sji.12819, PMID: 31448426

[50] Wu JQ Seay M Schulz VP Hariharan M Tuck D Lian J. Tcf7 is an important regulator of the switch of self-renewal and differentiation in a multipotential hematopoietic cell line. PLoS Genet. (2012) 8:e1002565. doi: 10.1371/journal.pgen.1002565, PMID: 22412390 PMC3297581

[51] Anjos SM Tessier M-C Polychronakos C. Association of the Cytotoxic T Lymphocyte-Associated Antigen 4 gene with type 1 diabetes: evidence for independent effects of two polymorphisms on the same haplotype block. J Clin Endocrinol Metabol. (2004) 89:6257–65. doi: 10.1210/jc.2004-0881, PMID: 15579786

[52] Ligers A Teleshova N Masterman T Huang W-X Hillert J. CTLA-4 gene expression is influenced by promoter and exon 1 polymorphisms. Genes Immun. (2001) 2:145–52. doi: 10.1038/sj.gene.6363752, PMID: 11426323

[53] Huang P-Y Guo S-S Zhang Y Lu J-B Chen Q-Y Tang L-Q. Tumor CTLA-4 overexpression predicts poor survival in patients with nasopharyngeal carcinoma. Oncotarget. (2016) 7:13060–8. doi: 10.18632/oncotarget.7421, PMID: 26918337 PMC4914341

[54] Omura Y Toiyama Y Okugawa Y Yin C Shigemori T Kusunoki K. Prognostic impacts of tumoral expression and serum levels of PD-L1 and CTLA-4 in colorectal cancer patients. Cancer Immunol Immunother. (2020) 69:2533–46. doi: 10.1007/s00262-020-02645-1, PMID: 32577816 PMC11027465

[55] Kamal AM Wasfey EF Elghamry WR Sabry OM Elghobary HA Radwan SM. Genetic signature of CTLA-4, BTLA, TIM-3, and LAG-3 molecular expression in colorectal cancer patients: implications in diagnosis and survival outcomes. Clin Biochem. (2021) 96:13–8. doi: 10.1016/j.clinbiochem.2021.06.007, PMID: 34217699

[56] Atanackovic D Luetkens T. Biomarkers for checkpoint inhibition in hematologic malignancies. Semin Cancer Biol. (2018) 52:198–206. doi: 10.1016/j.semcancer.2018.05.005 29775689

[57] Havel JJ Chowell D Chan TA. The evolving landscape of biomarkers for checkpoint inhibitor immunotherapy. Nat Rev Cancer. (2019) 19:133–50. doi: 10.1038/s41568-019-0116-x, PMID: 30755690 PMC6705396

[58] Fietz S Zarbl R Niebel D Posch C Brossart P Gielen GH. CTLA4 promoter methylation predicts response and progression-free survival in stage IV melanoma treated with anti-CTLA-4 immunotherapy (ipilimumab). Cancer Immunol Immunother. (2021) 70:1781–8. doi: 10.1007/s00262-020-02777-4, PMID: 33196890 PMC8139923

[59] Klümper N Ralser DJ Zarbl R Schlack K Schrader AJ Rehlinghaus M. CTLA4 promoter hypomethylation is a negative prognostic biomarker at initial diagnosis but predicts response and favorable outcome to anti-PD-1 based immunotherapy in clear cell renal cell carcinoma. J Immunother Cancer. (2021) 9:e002949. doi: 10.1136/jitc-2021-002949, PMID: 34446578 PMC8395367

